# Oral epigallocatechin-3-gallate for treatment of dystrophic epidermolysis bullosa: a multicentre, randomized, crossover, double-blind, placebo-controlled clinical trial

**DOI:** 10.1186/s13023-016-0411-5

**Published:** 2016-03-25

**Authors:** Christine Chiaverini, Coralie Roger, Eric Fontas, Emmanuelle Bourrat, Eva Bourdon-Lanoy, Christine Labrèze, Juliette Mazereeuw, Pierre Vabres, Christine Bodemer, Jean-Philippe Lacour

**Affiliations:** Reference Centre for Inherited Epidermolysis Bullosa, Archet 2 Hospital, Nice, France; INSERM, U1081, CNRS, UMR7284, Institute for Research on Cancer and Aging of Nice, University of Nice Sophia Antipolis, Nice, France; Department of Clinical Research and Innovation, University Hospital of Nice, Nice, France; Reference Centre of Rare Skin Diseases, MAGEC 5, Saint Louis Hospital, APHP, Paris, France; Reference Centre of Rare Skin Diseases, Pellegrin Hospital, Bordeaux, France; Reference Centre of Rare Skin Diseases, Larrey Hospital, Toulouse, France; Department of Dermatology, Bocage Hospital, Dijon, France; Reference Centre of Rare Skin Diseases, MAGEC, Necker Hospital, APHP, Institut Imagine, Paris, France; Université Paris Descartes - Sorbonne Paris Cité, Paris, France

**Keywords:** Dystrophic epidermolysis bullosa, Polyphenon, Green tea, Cathechin, Metalloproteinase

## Abstract

**Abstract:**

Recessive dystrophic epidermolysis bullosa (RDEB) is a rare genodermatosis with severe blistering. No curative treatment is available. Scientific data indicated that epigallocatechin-3-gallate (EGCG), a green tea extract, might improve the phenotype of RDEB patients. In a multicentre, randomized, crossover, double-blind, placebo-controlled clinical trial, we evaluated a 4-month oral EGCG treatment regimen in 17 RDEB patients. We found that EGCG treatment was not more effective than placebo in modified intention to treat and *per protocol* analysis (*n* = 16; *p* = 0.78 and *n* = 10; *p* = 1 respectively). Tolerance was good. Specific organizational and technical difficulties of controlled randomized double-blind trials in EB patients are discussed.

**Trial registration:**

US National Institutes of Health Clinical Trial Register (NCT00951964).

**Electronic supplementary material:**

The online version of this article (doi:10.1186/s13023-016-0411-5) contains supplementary material, which is available to authorized users.

Recessive dystrophic epidermolysis bullosa (RDEB) is a rare genodermatosis characterized by skin and mucosal fragility due to mutations in the *COL7A1* gene [1]. No curative treatment is available [2]. It has been shown that the level of activation of dermal metalloproteinases (MMP) could modulate the phenotype in RDEB patients [3–5] and that epigallocatechin-3-gallate (EGCG), a green tea extract [6–8], is able to regulate this activity *in vitro and ex vivo* [9].

We then evaluated the efficacy of oral EGCG to improve skin impairment in RDEB patients in a multicentre, randomized, crossover, double-blind, placebo-controlled clinical trial. The trial was approved by the local ethics committee and was registered in the Clinical Trial Register (NCT00951964). Patients of both sexes, over 2 years of age, with generalized severe or intermediate RDEB, confirmed by immunohistological analysis of skin biopsy, were recruited. Patients received treatment or placebo for 4 months, followed by a 2-month wash-out period and then by the other treatment for 4 months (Fig. 1). Dosage of EGCG treatment depended on the patient’s weight (from 400 to 800 mg a day) (Additional file 1, Supplementary Methods).

**Fig. 1:**
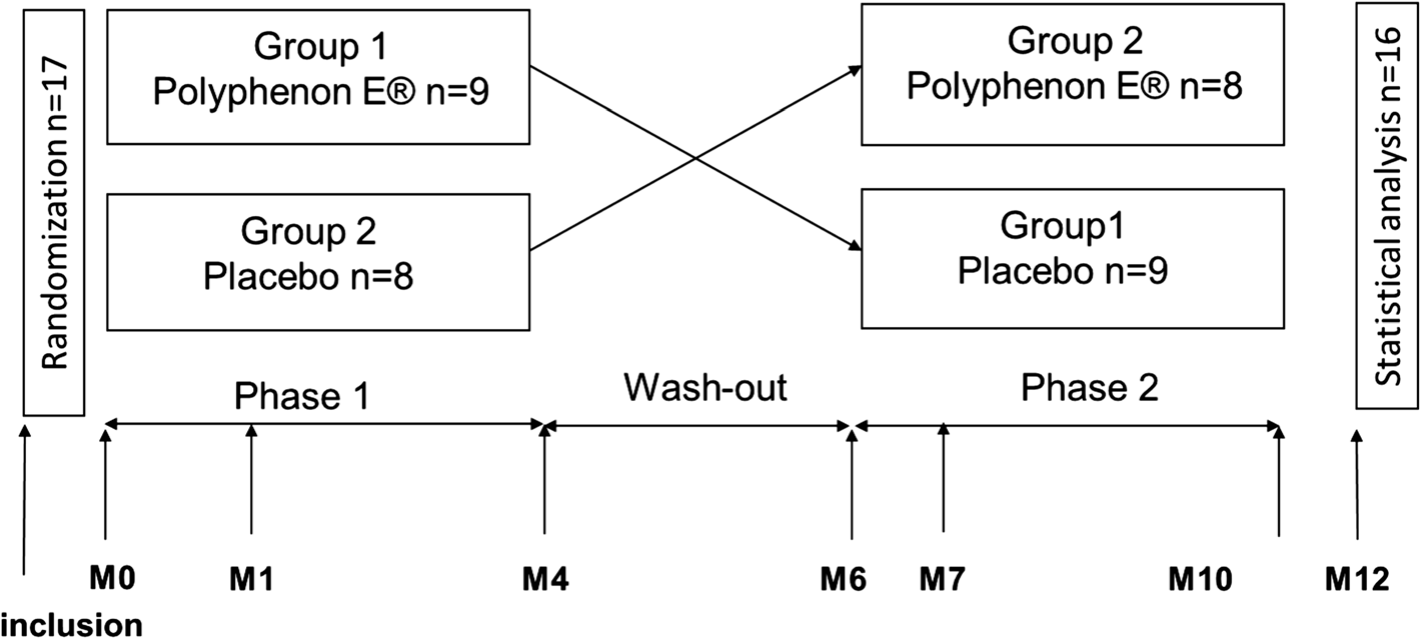
Study design. Each patient has a 4 month period of treatment separated by a 2 month period of wash-out

The main outcome was binary: *success*, defined as a decrease ≥ 20 % in the number of new blisters per day counted by patients at each dressing, upon 7 consecutive dressings before the initial and final visit of each treatment period, or *failure* of treatment. Secondary outcomes were the affected cutaneous surface area, the severity of mucosal impairment, skin fragility, itch and the mean duration of healing measured on 3 new blisters selected by patients in the first week of each period of treatment (Additional file 2: Table S1). Adverse events were collected by investigators at each visit. Assuming an expected success rate of 30 % in the EGCG group and 5 % in the placebo group, with 80 % power and 5 % type I error, we planned to include 22 patients. The main outcome was analysed in a modified intention to treat (mITT) and *per protocol*, secondary outcomes in a mITT.

## Findings

Seventeen patients were included in this study, mean age 19.4 years (±16.2 SD). One patient did not start treatment and was not included in the mITT population (*n* = 16). Only 10/16 patients were included in the *per protocol* analysis (available data for each visit in both treatment periods for the main outcome). Eight patients/16 (50 %) had a decrease of at least 20 % in the mean number of new blisters per day with EGCG and 5/16 (31 %) with placebo in the mITT analysis. This difference was not statistically significant (Prescott’s test, *p* = 0.78). Results were similar in *per protocol* analysis (*p* = 1). Analyses of secondary outcomes showed no difference between the 2 treatment periods (Table 1) despite a dramatic reduction of the mean duration of wound in the EGCG group (-14.62 days ± 18.76) compared to the placebo group (1.78 ± 14.65). Tolerance was good with 26 and 16 adverse events in the EGCG and placebo group respectively (*p* = 0.47) (Additional file 3: Table S2).

**Table 1:** Statistical analysis of secondary outcomes

Evolution of score (end of the period - beginning of the period)	Polyphenon E® Mean ± SD (*N*)	Placebo Mean ± SD (*N*)	*p* value
Surface area	- 4.07 ± 7.62 (12)	- 4.42 ± 9.84 (14)	0.93
Skin fragility	- 0.90 ± 2.46 (12)	- 0.64 ± 2.06 (14)	0.75
Mucosal involvement	0.55 ± 1.12 (8)	1.97 ± 1.64 (6)	0.07
Itch	- 1.17 ± 3.53 (12)	0 ± 2.16 (14)	0.38
Mean duration of wound healing (days)	-14.62 ± 18.76 (7)	1.78 ± 14.65 (9)	0.21

*N* number of patients, *SD* standard deviation

Generalized DEB is a rare and severe genodermatosis. Hence, evaluation of a new treatment in a controlled randomized and double-blind trial is challenging. In this study, even if fewer new blisters per day were observed in the EGCG arm as compared with the placebo and the mean duration of wound healing was shorter, we failed to show a statistically significant difference. These disappointing results can be explained by several limitations of our study. First, under-enrolment and the high rate of missing data for the main outcome are of important concern. Low enrolment is a major drawback for studies on all rare and severe diseases [10–13]. Indeed, despite the active involvement of the DEBRA France patients’ support group and the main French centres for EB care, together with the reimbursement of the patient’s travel expenses, only 17 patients instead of the 22 planned could be enrolled and only 10 completed the study. Shorter studies with less visits and/or home evaluation by a study nurse and/or international studies could improve the patient recruitment and protocol adherence. Moreover, factors influencing the severity of phenotype in DEB are complex and not only related to the MMP activity as recently shown [3, 14, 15]. Finally the high rate of therapeutic success in the placebo group is intriguing, but seems to be frequent in the few controlled versus placebo published studies on DEB [10–13]. The variable course of DEB, depending on numerous factors such as the weather, associated diseases and or/secondary complications or trauma, is well known. We tried to minimize the impact of these factors by counting the number of new blisters per day averaged on seven consecutive dressings before each visit. However other outcome measures like a validated EB severity score may be more relevant. Analysis of the inclusion date of each patient did not support an influence of seasonal variation. EGCG is a potentially interesting and safe treatment for DEB patients. An international randomized, double-blinded and placebo-controlled trial with targeted subpopulation is necessary.

## Additional files

Additional file 1: Supplementary methods. (DOC 22 kb) Additional file 2: Primary and secondary outcomes assessments. (DOC 31 kb) Additional file 3: Detailed adverse events for EGCG and placebo treatment. * indicates the adverse events reported as severe by investigators. (DOC 41 kb)

## Abbreviations

DEB: dystrophic epidermolysis bullosa
EGCG: epigallocatechin-3-gallate
mITT: modified intention to treat
MMP: metalloproteinases
RDEB: recessive dystrophic epidermolysis bullosa
SD: standard deviation
