# Supplementary material for: Oral epigallocatechin-3-gallate for treatment of dystrophic epidermolysis bullosa: a multicentre, randomized, crossover, double-blind, placebo-controlled clinical trial
Source: Orphanet J Rare Dis. 2016 Mar 25;11:31. doi: 10.1186/s13023-016-0411-5 (PMC4807580; doi:10.1186/s13023-016-0411-5)
Supplement: Additional file 1: — Supplementary methods. (DOC 22 kb) [file 13023_2016_411_MOESM1_ESM.doc]

Additional file 1: Supplementary methods

Study design: After inclusion, patients were centrally randomized, through fax exchange with methodological centre (computer generated list with permutation block (size=4) method, Department of Clinical Research, Nice University Hospital), in a 1:1 ratio to receive treatment or placebo for 4 months, followed by a 2-month wash-out period. Patients then received the other treatment for 4 months (Figure 2: study design). Each patient had 8 follow-up visits with medical examination: at selection, at inclusion (M0), and at 1, 4, 6, 7, 10 and 12 months.

Treatment administration: EGCG was conditioned in 200 mg capsules as was the placebo. As most RDEB patients cannot swallow capsules, they were asked to open the capsules and dilute their content in water and drink it immediately. Patients with a gastrostomy were allowed to use this route to take the treatment.
