# Supplementary material for: Oral epigallocatechin-3-gallate for treatment of dystrophic epidermolysis bullosa: a multicentre, randomized, crossover, double-blind, placebo-controlled clinical trial
Source: Orphanet J Rare Dis. 2016 Mar 25;11:31. doi: 10.1186/s13023-016-0411-5 (PMC4807580; doi:10.1186/s13023-016-0411-5)
Supplement: Additional file 2: — Primary and secondary outcomes assessments. (DOC 31 kb) [file 13023_2016_411_MOESM2_ESM.doc]

Additional file 2, Table S1: Primary and secondary outcomes assessments.

|  | | Assessment | Evaluator | Date |
| --- | --- | --- | --- | --- |
| Main outcome | | Mean number of new blisters counted at each dressing, calculated upon 7 consecutive dressings before visit | Patient or parents | M0, M4, M6 and M10 |
| Secondary outcomes | affected cutaneous surface area | Body diagram | Investigator |  |
|  | Skin fragility | Visual analogic scale | Patient or parents |  |
|  | Mucosal involvement | Visual analogic scale | Patient or parents |  |
|  | Pruritus | Visual analogic scale | Patient or parents |  |
|  | Duration of healing | Mean duration of complete healing of 3 new blisters selected by patients in the first week of each period of treatment | Patient or parents | M4 and M10 |
