# Supplementary material for: Oral epigallocatechin-3-gallate for treatment of dystrophic epidermolysis bullosa: a multicentre, randomized, crossover, double-blind, placebo-controlled clinical trial
Source: Orphanet J Rare Dis. 2016 Mar 25;11:31. doi: 10.1186/s13023-016-0411-5 (PMC4807580; doi:10.1186/s13023-016-0411-5)
Supplement: Additional file 3: — Detailed adverse events for EGCG and placebo treatment. * indicates the adverse events reported as severe by investigators. (DOC 41 kb) [file 13023_2016_411_MOESM3_ESM.doc]

Additional file 3, Table S2: Detailed adverse events for EGCG and placebo treatment.

| **List of adverse events** | **Placebo** | **EGCG** | **P value** |
| --- | --- | --- | --- |
| Diarrhea | 2 | 2 | 0.256 |
| Gastroenteritis | 0 | 3 |
| Vomiting | 0 | 1 + 1 |
| Esophageal pain | 2 | 0 |
| Esophageal blister* | 0 | 1 |
| Odynophagia * | 0 | 1 |
| Constipation | 0 | 1 |
| Anal fissure | 0 | 1 |
| Skin superinfection | 1 | 2 |
| Flare of blisters | 1 | 0 |
| Flare of atopic dermatitis | 0 | 1 |
| Pruritus | 0 | 1 |
| Ainhum* | 0 | 1 |
| Upper respiratory tract infection (one was declared as severe*) | 5 | 3 |
| Bronchitis | 0 | 2 |
| Asthenia | 0 | 1 |
| Epidermoid carcinoma* | 2 | 1 |
| Fall | 1 | 1 |
| Vaginal inflammation | 1 | 0 |
| Herpes | 0 | 1 |
| Lumbago | 0 | 1 |
| Surgery of the foot* | 1 | 0 |
| **TOTAL** | **16** | **26** |
| Severe adverse events | 4 | 4 | 0.351 |
| Possible responsibility of the treatment | 5 | 3 |  |

* indicates the adverse events reported as severe by investigators. Adverse events reported with possible responsibility of the treatment are underlined.
